# Prospective Association of High Effort and Low Reward Imbalance at Work with Risk of Diabetes: A Cohort Study in US Workers

**DOI:** 10.1007/s12529-023-10168-z

**Published:** 2023-04-11

**Authors:** Natalia Wege, Johannes Siegrist, Jian Li

**Affiliations:** 1 Institute of General Practice, Centre for Health and Society, Medical Faculty, Heinrich Heine University Düsseldorf, PO Box 10 10 07, D-40001 Düsseldorf, Germany; 2 Institute of Medical Sociology, Faculty of Medicine, University of Düsseldorf, Düsseldorf, Germany; 3 Department of Environmental Health Sciences, Fielding School of Public Health, School of Nursing, University of California, Los Angeles, Los Angeles, USA

**Keywords:** Type 2 diabetes, Psychosocial stress, Effort-reward imbalance model, Workers

## Abstract

**Background:**

The contribution of psychosocial stress in the workplace to development of type 2 diabetes mellitus (T2DM) is not well investigated. As most studies were conducted in Europe, a further test from the USA seems well justified. The objective of the current investigation was to examine prospective associations of work stress based on the effort-reward imbalance model with risk of T2DM in a national sample of US workers.

**Method:**

Using data from the national population-based Mid-life in the United States (MIDUS) study with a prospective cohort design and a 9-year follow-up period, the effects of a ratio combining data on effort and reward at work (ER ratio) at baseline on risk of T2DM at follow-up were examined in 1493 workers who were free from diabetes at the baseline survey, applying multivariable Poisson regression analysis.

**Results:**

During the follow-up, 109 individuals (7.30%) reported onset of diabetes. The analyses demonstrated a significant association between continuous data of the E-R ratio and risk of diabetes (RR and 95% CI = 1.22 [1.02, 1.46]), after adjustment for modifiable and non-modifiable risk factors at baseline. A dose-dependent response was observed with trend analysis when using quartiles of the E-R ratio.

**Conclusion:**

In the US workers, high effort in combination with low reward at work was significantly associated with elevated risk of T2DM 9 years later. The risk profiles of diabetes should be adapted in consideration of psychosocial work environment and taken into account by conceptualizing prevention programs of chronic non-communicable diseases.

## Introduction

With a worldwide increase of obesity in most Western countries, the prevalence of metabolic disorders, and specifically type 2 diabetes mellitus (T2DM), provides a challenge for health and social policy [1]. Recent research supports the notion that chronic psychosocial stress is a further risk factor of diabetes in adults, operating via enhanced unhealthy behaviors and via psychobiological pathways of activated stress axes within the organism [2]. Working conditions provide an important opportunity of studying the chronicity of stressful experience. Accordingly, several prospective cohort studies examined an association of chronic psychosocial stress at work with risk of incident diabetes. In majority, these studies used the demand-control model [3] or the effort-reward imbalance model [4] to define and assess chronic psychosocial stress at work. The former model maintains that job task profiles characterized by high quantitative demand and a low degree of control (in terms of decision latitude and skill discretion) provoke continued strain reactions among exposed workers, increasing their susceptibility of developing a stress-related disorder [5]. As a complementary approach, the effort-reward imbalance model focuses on critical aspects of the work contract, where an imbalance between high effort spent and low rewards received in turn elicits strong negative emotions and associated psychobiologic stress responses with adverse long-term effects on health [6]. Importantly, rewards at work include money, promotion prospects, job security, and appreciation of achieved work. Two recent systematic reviews with meta-analyses confirm that in a majority of prospective cohort studies, both concepts of chronic psychosocial stress at work were associated with as a moderately increased risk of diabetes [7, 8]. The effect size was generally larger among working women than among working men. Yet, as most studies were conducted in Europe, and as some inconsistency of findings persists, a further test of this hypothesis seems well justified, using data from the USA. While one recent study explored this question among older workers in this country [9], the current investigation sets out to analyze the association of effort-reward imbalance at work with risk of incident diabetes in a middle-aged working sample, based on data from the national population-based Mid-life in the United States (MIDUS) study with a prospective cohort design and a 9-year follow-up period.

## Methods

### Study Population

Data from the 2nd and 3rd surveys of the Mid-life in the United States (MIDUS) study were utilized. A detailed description of MIDUS is published elsewhere [10]. In brief, the 2nd survey was carried out from 2004 to 2006, while the third occurred from 2013 to 2014, providing a follow-up period of approximately 9 years. Out of 4963 participants at baseline, 2313 reported that they were working. Among them, 2211 workers (95.6%) had complete data on relevant variables at baseline. During the 3rd survey, 1738 participants were followed up (follow-up rate = 78.6%). We excluded 102 participants who reported diabetes at baseline, and 143 individuals who did not offer valid information on diabetes at follow-up, yielding a final sample size of 1493 for the current analyses. All participants provided written informed consent. This study was reviewed and approved for exemption by the University of California, Los Angeles, Institutional Review Board (IRB#22–000604).

### Measures

A 17-item scale was used to measure the two extrinsic scales ‘effort’ and ‘reward’ of the work stress model at baseline, including 10 items for effort and 7 items for reward (Cronbach’s alpha coefficients for these validated proxy measures were 0.74 for effort and 0.76 for reward [11]). As a summary index of the balance/imbalance between effort and reward, an E-R ratio was calculated by dividing the sum scores of effort items by the sum scores of reward items, weighted by the number of items, according to an established procedure [4]. The E-R ratio was operationalized across separate regression models alternatively as a categorical measure, using quartiles as cut-points, and as a continuous measure (standardized Z-score as well. During both surveys, participants were asked the following two questions: “in the past 12 months, have you experienced or been treated for diabetes or high blood sugar”, “during the past 30 days, have you taken prescription medicine for diabetes”. T2DM was defined as ‘yes’ to either of the above questions. This approach has been applied in previous MIDUS publication [12]. At baseline, information on sociodemographic factors and health-related behaviors were collected, including age, sex, race, marital status, education, household income, current smoking, alcohol consumption, and physical exercise (see also [8, 10]).

### Statistical Analysis

Descriptive statistics were generated as the first step: means and standard deviations (SDs) were investigated for continuous variables, and relative frequencies were examined for categorical variables. Then prospective associations of the E-R ratio at baseline with risk of T2DM at follow-up were estimated using Poisson regression with a robust error variance, and the results were expressed as risk ratios (RRs) with 95% confidence intervals (CIs) [13]. To exclude possible influence of known modifiable and non-modifiable risk factors, in particular age, sociodemographic factors, physical inactivity, tobacco use, and alcohol consumption [14], multivariable regression models were conducted in three steps. Model I was adjusted for sex and age, model II additionally was adjusted for race, marital status, educational attainment, and annual household income. Model III included additional adjustment for the health-related behaviors of smoking, alcohol consumption, and physical activity. All analyses were conducted using the SAS 9.4 software package.

## Results

Table 1 displays the main characteristics of the study sample. The mean age was 51 years, with similar proportions of women and men. A majority was white, and those with higher education were over-represented. In terms of healthy lifestyle, the sample was rather protected from high risk. Overall, the mean level of stressful work was moderate, if compared with samples from other countries.

**Table 1:** Characteristics of study subjects at baseline (N, %)

Variables		
Age (years)	Mean (SD)	51.15 (9.08)
Sex	Men	710, 47.56%
	Women	783, 52.44%
Race	White	1394, 93.37%
	Black	39, 2.61%
	Others	60, 4.02%
Marital status	Married	1108, 74.21%
	Never married	132, 8.84%
	Others	253, 16.95%
Education	High school or less	363, 24.31%
	Some college	412, 27.60%
	University or more	718, 48.09%
Annual household income (US $)	< 60,000	547, 36.64%
	60,000–99,999	481, 32.22%
	≥ 100,000	465, 31.14%
Current smoking	No	1297, 86.87%
	Yes	196, 13.13%
Alcohol consumption	No to moderate	1453, 97.32%
	Heavy	40, 2.68%
Physical exercise	Low	330, 22.10%
	Moderate	512, 34.29%
	High	651, 43.61%
E-R ratio	Mean (SD)	0.72 (0.22)

Results on the main hypothesis are given in Table 2. With each increase of 1 SD of the E-R ratio, a significantly increased risk of incident diabetes is observed. Furthermore, a dose–response relationship is apparent if the quartiles of the E-R ratio are used as predictors of incident diabetes risk. Compared to the group with no stress at work (lowest quartile), those scoring in the highest quartile of the E-R ratio exhibit an RR of 2.04 (with confidence interval beyond 1.0). Of interest, this effect is nor substantially modified when adjusting for two sets of confounding factors (RR of 1.80 for model 2 and RR of 1.74 for model 3).

**Table 2:** Prospective associations of work stress with risk of diabetes (RRs and 95% CIs) (*N* = 1493)

Variables	New cases of diabetes at follow-up (*N*, %)	Model I	Model II	Model III
E-R ratio continuous				
Increase per SD	109 (7.30%)	1.30 (1.10, 1.54)**	1.24 (1.04, 1.48)*	1.22 (1.02, 1.46)*
E-R ratio divided into quartiles				
Low quartile	23 (6.02%)	1.00	1.00	1.00
Medium–low quartile	23 (6.28%)	1.34 (0.84, 2.15)	1.33 (0.83, 2.14)	1.27 (0.79, 2.04)
Medium–high quartile	28 (7.51%)	1.77 (1.08, 2.91)*	1.61 (0.98, 2.64)	1.50 (0.92, 2.46)
High quartile	35 (9.41%)	2.04 (1.23, 3.38)**	1.80 (1.08, 3.01)*	1.74 (1.04, 2.90)*
* p* for trend		0.0033	0.0183	0.0277

Model I: adjustment for age and sex at baseline

Model II: model I + additional adjustment for race, marital status, educational attainment, and household income at baseline

Model III: model II + additional adjustment for smoking, alcohol consumption, and physical exercise at baseline

*CI* confidence interval, *RR* risk ratio, *SD* standard deviation

Poisson regression, *p < 0.05; **p < 0.01

## Discussion

This investigation corroborates previous findings from prospective studies indicating that an adverse psychosocial work environment, assessed in terms of the effort-reward imbalance model, is associated with a moderately increased risk of incident diabetes in US workers. The effect size of this estimate in the fully adjusted multivariable model (RR = 1.22; 95% CI 1.02; 1.46) is comparable to other studies, for instance, one older cohort in the USA (HR = 1.33; 95% CI 1.04; 1.69) [9] and the one recent meta-analysis of cohort studies (RR = 1.24,95% CI 1.08; 1.42) [7]. In general, this effect is somewhat stronger among working women than men, but in the current analysis, gender differences were not statistically significant (data not shown). This report broadens the knowledge on associations of stressful work with risk of diabetes as previous research was mainly based on the demand-control model [11, 15]. In a comparative recent meta-analysis of the two work stress models that addressed additionally risk of bias, significantly elevated risk ratios were of similar size (1.16 for the demand-control model,1.24 for the effort-reward imbalance model [7]. Importantly, this meta-analysis concluded that effects of single components of these models, when analyzed separately, did not show significant associations with T2DM. Based on this evidence, analyses of the current study were restricted to the summary measure of the E-R ratio.

Despite the strengths of a prospective study design, the use of a validated exposure measure, the inclusion of a set of confounding variables, and the analysis of continuous and categorical effects on the outcome criterion, this report suffers from several limitations. First, concerning assessment of diabetes, data based on self-report did not separate T1DM from T2DM clearly. However, participants with diabetes were excluded at baseline assessment of this study population. As incidence data referred to a middle-aged population, likelihood of T1DM incidence would be quite low. With respect to the validity of self-reported information on diabetes, satisfactory values of specificity (84–97%) and sensitivity (55–80%) were reported using multiple reference definitions in community-based population [16]. As a second limitation, the study protocol did not include the original scales of the effort-reward imbalance model, but an extensive psychometric validation confirmed a satisfactory comparison with the original approach [11]. Moreover, for the reason mentioned, we did not additionally analyze the effects of the two extrinsic single-model components, and no data were available on the model’s intrinsic component of over-commitment. It is therefore possible that the effect size was slightly underestimated. Third, the dataset did not include all known risk factors of diabetes, thus leaving uncertainty about residual confounding. Unfortunately, no data on body weight was available, a variable of interest not only as a confounder, but in addition as a potential mediator in the association of stressful work with T2DM. These limitations underline the need for further comprehensive investigations into psychosocial occupational determinants of metabolic risks enriched by biological parameters and contextual data. Finally, our results are based on the national cohort of US workers and could be not generalized for the whole world population. However, taking into account estimated resident population of the USA of 334,265,650 [17] and higher rates of diabetes, our results are still of practical significance.

Despite these restrictions, the current findings strengthen the consistency of evidence of a health-adverse effect due to exposure to psychosocial adversity at work. Jobs characterized by an imbalance between high cost and low gain, as well as jobs defined by high demands and low control, deserve systematic monitoring and respective preventive efforts in the context of targeted worksite health promotion programs.

## Data Availability

All relevant data from the Midlife in the United States (MIDUS) Study can be downloaded for free at http://midus.wisc.edu/data/index.php.

## Declarations

### Statement Regarding the Welfare of Animals

This article does not contain any studies with animals performed by any of the authors.
